# Extravasation of Urine into the Corpus Spongiosum and Corpora Cavernosa Penis, Producing Mortification of Those Parts, and the Death of the Patient

**Published:** 1827-09

**Authors:** 

**Affiliations:** St. George's Hospital


Case III. *Extravasation of Urine into the Corpus Spongiosum and Corpora Cavernosa Penis, producing Mortification of those Parts, and the Death of the Patient.* Treated at St. George's Hospital, by Mr. Jeffreys.

In the more ordinary cases of extravasation of urine, the rupture of the urethra takes place between the bulb and the bladder, and the urine is forced into the cellular membrane surrounding that part of the canal. The symptoms produced by this accident are of a most formidable description, but, by prompt and skilful management, the life of the patient may frequently be saved. Sometimes the breach takes place in the anterior part of the urethra, and the urine escapes into the corpus spongiosum; more rarely it gets also into the cells of the corpora cavernosa: in both cases mortification of the parts is sure to follow, and the patient seldom or never recovers. The subjoined case affords an example of this accident, and fully illustrates the uncontrollable ravages produced by urine, when effused into the cells of the corpora cavernosa and corpus spongiosum.

William Willis, forty years of age, was admitted into St. George's Hospital, Friday, April 20th, 1827, with swelling and mortification of the penis, the consequence of an extravasation of urine; of which he gave the following history:—In the summer of 1826, he first observed that he had a difficulty in making water, and that it came away in a smaller stream than formerly, and at more frequent intervals. Towards Christmas these symptoms became more severe, and were accompanied by a gleety discharge, and at times he could only void his urine in drops, with much pain and straining. Latterly he had been subject to attacks of fever, with rigors, &c. The last of these had occurred about eight days ago, at which time there came on a constant dribbling of the urine, with swelling, pain, and inflammation of the penis. These symptoms got daily worse, and on Wednesday (two days previous to his admission) a small black spot appeared on the glans penis, near to the frœnum. This had spread with great rapidity, and now occupied more than two-thirds of the glans penis, and a considerable portion of the under part of the prepuce and corpus spongiosum. The penis, anterior to the scrotum, was prodigiously swollen, and the integuments covering it were of a deep orange-red colour, and much distended. He complained of great pain, and his urine dribbled away incessantly; but he said there had been no complete stoppage. He had a pale anxious countenance, a brown furred tongue, and a good deal of fever. The mischief appeared to be confined to the anterior part of the penis, the scrotum and perineum being entirely devoid of swelling and pain; and there could be no doubt, from the condition and appearance of the parts, that a breach had been effected in the urethra, a little way behind its termination in the glans penis.

With the approbation of Mr. Brodie and Mr. Rose, who happened to be in the hospital, I made an incision along the left side of the penis, where a strong sensation of fluctuation led to the expectation of finding a urinary abscess, and carried it through the ligamentous theca of the corpus cavernosum; the cellular membrane was in a state of slough, and loaded with urine; and the internal structure of the corpus cavernosum was of a dark colour, nearly resembling its natural appearance; but no distinct abscess was met with. Another incision was then carried through the mortified portion of the prepuce and corpus spongiosum, nearly to the scrotum, by which the urethra, also in a state of sphacelus, was exposed; and an attempt was made to pass a flexible gum catheter into the bladder; but neither that nor any other instrument could be got further than the membranous part of the urethra. A third incision was therefore made through the perineum into that portion of the canal, in order that a free and easy passage might be given for the discharge of the urine from the bladder.

The penis was enveloped in a large stale-beer poultice; and he was directed to take immediately ten grains of the Dover's powder, and afterwards, at intervals of four hours, a saline draught, containing half a drachm of the Sulphate of Magnesia, and three grains of the Pulv. Ipecac. comp. Beef-tea and arrow-root to be taken frequently.

In the evening, he was in all respects much relieved. He had enjoyed some refreshing sleep; had voided his urine very freely through the wound in the perineum, a small portion of it having escaped each time by the sloughy opening in the corpus spongiosum, and he complained less of pain in the parts. Pulse ninety-six; tongue dry in the centre.

He slept well in the night, and the next day (April 21st,) the pain and swelling of the penis were considerably reduced; he made water freely by the perineum, and said he felt much more comfortable. Not having had any stool, he took an ounce of Castor-oil early in the morning, which procured one plentiful evacuation; after which he was allowed four ounces of port-wine in the day; and was ordered the following draught every four hours:

℞. Decoct. Cinchon., Mist. Camph. āā ℥ vj.; Tinct. Opii gtt. v. M.

In the afternoon, he had two more purging stools from the oil.

April 22d.—Had slept in the night, but was occasionally delirious. He was more heated and feverish; had a dry, furred tongue, with much thirst; and his pulse was 100 in the minute. The sloughs were beginning to separate from the integuments at the under part of the penis.

He was ordered to omit the wine and bark draught, and to take Ol. Ricini ℥ ss.; and afterwards three spoonsful of the following mixture every six hours:—℞. Mist. Camph., Liq. Ammon. Acet. āā ℥ iv.; Vin. Antimon. Tart. ʒij. M.

23d.—Had been light-headed in the early part of the night, but his bowels had acted freely, and he was not so feverish. The whole of the glans penis was in a state of mortification, with the exception of a small narrow portion of the corona glandis, about a line in width: at this part a line of separation had begun, and the sloughs at the under part of the penis were more loose.

24th.—A light erysipelatous redness had appeared about the perineum and the inside of the thighs; his pulse was not so strong, and he seemed to be more weak and languid.

He was ordered some porter, and the following draught:—℞. Infusi Rosæ ℥ jss.; Tinct. Cinch. comp. ʒj.; Magn. Sulph. ʒ ss.; Syrupi ʒss. M. fiat haustus ter in die sumendus.

25th.—The separation of the mortified parts from the integuments was completed, leaving the latter in a sound and healthy state, but entirely detached from the corpora cavernosa and corpus spongiosum, which were in a complete state of gangrene and slough throughout their whole extent.

On the 26th, the erysipelas had spread to a small extent over the left buttock, upon which a few slight vesications had formed. The mortification of the corpora cavernosa appeared to have reached to each crus penis. He was much weaker; had a pale clammy countenance; tongue dry and furred; pulse 120.

Porter, wine, arrow-root, &c. were given at short intervals; and he was directed to take two grains of the Sulphate of Quinine every four hours.

On the 27th, he was much in the same state, and his skin covered with a profuse cold perspiration.

On the 28th, the erysipelas had nearly disappeared from the nates, and was spreading, of a pale colour, and without vesications, over the groins. The mortified corpora cavernosa and corpus spongiosum were entirely separated from the arch of the pubes and the scrotum, but adhered firmly to the parts below. A small portion of urine oozed out over the front of the scrotum every time he made water, but the greater proportion was voided freely in a stream by the opening in the perineum.

On the morning of the 29th, he died.

*Dissection.*—The corpora cavernosa, quite down to their origin from the ischia, and the corpus spongiosum, including the bulb, were completely black and mortified: no vestige of their natural structure remained. The coats of the bladder were more than half an inch thick; its internal coat, which was much corrugated, was of a dark colour, and covered with patches of coagulable lymph, closely adhering to its surface. The urethra had been cut into at the membranous part, from whence to the bladder it was quite pervious. Upon its inner surface were patches of coagulable lymph, like those in the bladder.

